# Case Report: Diagnosis of Late Spontaneous Intraocular Lens Dislocation on Point-of-care Ultrasound

**DOI:** 10.5811/cpcem.2021.3.52208

**Published:** 2021-07-27

**Authors:** Alexandra Pizarro, Thompson Kehrl

**Affiliations:** WellSpan York Hospital, Department of Emergency Medicine, York, Pennsylvania

**Keywords:** Intraocular lens, spontaneous, dislocation, point-of-care ultrasound, and POCUS

## Abstract

**Introduction:**

Spontaneous intraocular lens (IOL) dislocation is a rare, but serious, complication following cataract surgery.

**Case Report:**

We report a case of patient with a remote history of cataract surgery presenting to the emergency department with monocular blurred vision. Ocular point-of-care ultrasound (POCUS) facilitated diagnosis of a late spontaneous IOL dislocation.

**Discussion:**

Prosthetic IOL dislocations are being reported with increasing frequency. Prompt recognition of IOL dislocation is essential to prevent secondary complications, including acute angle-closure glaucoma and retinal detachment, which can result in permanent vision loss.

**Conclusion:**

Point-of-care ultrasound is a rapid, noninvasive imaging modality for early detection of IOL dislocation to help guide management, improve patient outcomes, and mitigate long-term sequelae.

## INTRODUCTION

Between 2007–2010, the US Centers for Disease Control and Prevention reported an average of 2.4 million ocular-related emergency department (ED) visits annually.^1^ Point-of-care ultrasonography (POCUS) is a non-invasive diagnostic tool that provides direct visualization of ocular structures. The use of POCUS in the ED has evolved and expanded in recent years to aid in the diagnosis of time-sensitive ocular conditions, including retinal detachment, vitreous hemorrhage, and intraocular lens (IOL) dislocation.^2^

## CASE REPORT

A 63-year-old male with history of retinal detachment and remote history of bilateral cataract surgery presented to the ED with blurry vision in his right eye. He described sudden-onset visual disturbance “like a curtain dropping” while walking earlier in the day. His vision transiently improved with bending over but would blur again when he stood upright. He denied any ocular trauma, eye pain or swelling, discharge, headache, or other focal neurological complaints. His past medical history included coronary artery disease, diabetes mellitus, hyperlipidemia, and hypertension.

On physical exam, pupils were equal and reactive to light bilaterally. Extraocular movements were intact. Visual acuity was 20/100 in the right eye and 20/30 in the left eye. Slit lamp exam of the right eye revealed a deep, quiet anterior chamber, but a lack of pupillary reflection and inability to visualize the retina.

Point-of-care ultrasound of the affected eye was performed in the sagittal and transverse anatomic planes using a high-frequency linear transducer. The prosthetic lens was visualized in the posterior chamber with the temporal side haptic still adherent to the lens capsule consistent with an IOL dislocation (Video, Image), whereas an appropriately positioned prosthetic lens would appear as a hyperechoic curvilinear structure within the lens capsule posterior to the iris. The patient was then asked to perform the six cardinal positions of gaze to maximize visualization of the ocular structures. There was no evidence of retinal detachment, vitreous detachment, or vitreous hemorrhage. The case was discussed with his ophthalmologist, and the patient underwent urgent operative repair of the dislocated lens as an outpatient.

## DISCUSSION

We report a unique case of a late spontaneous posterior IOL dislocation diagnosed by POCUS in an ED patient with remote history of cataract surgery. Cataract surgery involves removal of the natural cataractous lens and insertion of an artificial lens into the capsular bag. Intraocular lens dislocation is a rare complication of cataract surgery that often requires repeat surgical intervention.^3^–^5^

Intraocular lens dislocations occur in a bimodal distribution. Early IOL dislocation occurs within the initial three-month postoperative period, secondary to improper IOL fixation to the capsular bag or instability of the capsular bag resulting in zonular rupture. Late IOL dislocation occurs more than three months postoperatively, secondary to progressive zonular weakness and capsular bag contraction. Incidence of late IOL dislocations ranges between 0.05–3.0%.^6^ Rates of IOL dislocation increase over time, with a cumulative risk of 0.1% at five years, 0.2% at 15 years, and 1.7% at 25 years.^7^ The incidence of late IOL dislocations has been increasing and some studies suggest that this may be a result of an increasing life expectancy and longer life span of current lens materials.^6^ Other studies suggest that this trend is multifactorial, related to greater numbers of cataract surgeries performed annually, variations in surgical approach, lens material, and other risk factors such as zonular weakness.^5^, ^8^

Dislocation of the prosthetic lens through a defect or tear in the capsular bag is referred to as in-the-bag dislocation, whereas destabilization and migration of the entire capsular bag constitutes an out-of-the-bag dislocation. Severity ranges from partial dislocations causing mild phacodonesis (vibration/tremulousness of the lens with eye movement) to complete dislocations with migration of the lens into the anterior or posterior chambers.^3^, ^9^ Displacement of the lens into the anterior chamber can result in acute angle-closure glaucoma, and displacement into the posterior chamber can cause retinal detachment. Other serious complications associated with IOL dislocation include corneal decompensation, lens perforation or dislocation, vitreous detachment, and corneal or macular edema.^7^, ^10^

**CPC-EM Capsule**

What do we already know about this clinical entity?

*Point-of-care ultrasonography is a rapid, non-invasive imaging modality that can provide direct visualization of ocular structures to aid in the diagnosis of time-sensitive ophthalmologic conditions*.

What makes this presentation of disease reportable?

*Intraocular lens dislocation is a rare, but serious, complication following cataract surgery that is being reported with increased frequency*.

What is the major learning point?

*Prompt recognition of intraocular lens dislocation is essential in preventing secondary complications, such as acute angle-closure glaucoma and retinal detachment, which can result in permanent vision loss*.

How might this improve emergency medicine practice?

*Point-of-care ultrasound is an excellent first-line imaging modality for ocular complaints that can help guide management, improve patient outcomes, and mitigate long-term sequelae*.

Patients with IOL dislocation typically present with visual changes, including blurred vision, double vision, or the ability to visualize the edge of lens implant. An IOL dislocation can be diagnosed using multiple imaging modalities, including ultrasonography, computed tomography, and magnetic resonance imaging. Point-of-care ultrasound is a rapid, noninvasive modality that provides direct visualization of the ocular structures and is an excellent first-line choice for ocular complaints in the ED.^11^ Intraocular lens dislocation typically requires surgical repositioning or replacement of the prosthetic lens. Urgency of treatment largely depends on the type of IOL, the site of IOL dislocation, and coexisting ocular pathology.^12^

## CONCLUSION

Complications following cataract surgery, such as IOL dislocation, are being reported with increased frequency. Prompt recognition of IOL dislocation is essential in preventing complications that can result in permanent loss of vision. Point-of-care ultrasound provides a rapid, noninvasive method for early detection of IOL dislocation to help guide management, improve patient outcomes, and mitigate long-term sequelae.

## Supplementary Information

Video Ultrasound clip showing intraocular lens dislocation (arrow) into the posterior chamber. The prosthetic lens appears as a hyperechoic curvilinear structure in the posterior chamber with the temporal side haptic still adherent to the lens capsule, whereas an appropriately positioned prosthetic lens would appear within the lens capsule posterior to the iris.

## Figures and Tables

**Image:**
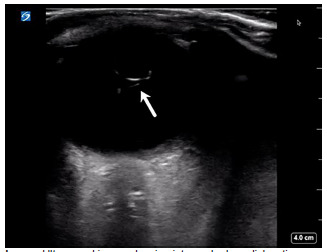
Ultrasound image showing intraocular lens dislocation (arrow) into the posterior chamber.
